# Improving the Quantitative Analysis of Breast Microcalcifications: A Multiscale Approach

**DOI:** 10.1007/s10278-022-00751-3

**Published:** 2023-02-23

**Authors:** Chrysostomos Marasinou, Bo Li, Jeremy Paige, Akinyinka Omigbodun, Noor Nakhaei, Anne Hoyt, William Hsu

**Affiliations:** 1 Medical & Imaging Informatics, Department of Radiological Sciences, David Geffen School of Medicine at UCLA, 924 Westwood Blvd, Ste 420, Los Angeles, 90024 USA; 2 Department of Radiological Sciences, David Geffen School of Medicine at UCLA, Los Angeles, 90095 CA USA; 3 Department of Computer Science, UCLA Samueli School of Engineering, Los Angeles, 90095 CA USA

**Keywords:** Breast cancer, Full-field digital mammography, Microcalcifications, Segmentation

## Abstract

Accurate characterization of microcalcifications (MCs) in 2D digital mammography is a necessary step toward reducing the diagnostic uncertainty associated with the callback of indeterminate MCs. Quantitative analysis of MCs can better identify MCs with a higher likelihood of ductal carcinoma in situ or invasive cancer. However, automated identification and segmentation of MCs remain challenging with high false positive rates. We present a two-stage multiscale approach to MC segmentation in 2D full-field digital mammograms (FFDMs) and diagnostic magnification views. Candidate objects are first delineated using blob detection and Hessian analysis. A regression convolutional network, trained to output a function with a higher response near MCs, chooses the objects which constitute actual MCs. The method was trained and validated on 435 screening and diagnostic FFDMs from two separate datasets. We then used our approach to segment MCs on magnification views of 248 cases with amorphous MCs. We modeled the extracted features using gradient tree boosting to classify each case as benign or malignant. Compared to state-of-the-art comparison methods, our approach achieved superior mean intersection over the union (0.670 ± 0.121 per image versus 0.524 ± 0.034 per image), intersection over the union per MC object (0.607 ± 0.250 versus 0.363 ± 0.278) and true positive rate of 0.744 versus 0.581 at 0.4 false positive detections per square centimeter. Features generated using our approach outperformed the comparison method (0.763 versus 0.710 AUC) in distinguishing amorphous calcifications as benign or malignant.

**Supplementary Information:**

The online version contains supplementary material available at 10.1007/s10278-022-00751-3.

## Introduction

Breast cancer is the most common cancer in women, accounting for 12% of cancer cases worldwide [1]. Studies have shown that early detection using mammography reduces breast cancer mortality [2]. In many countries, screening programs have been established with sensitivity levels ranging between 80 and 95% [3, 4]. However, screening also results in false positive outcomes, leading to patient anxiety, unnecessary biopsies, and the identification of clinically insignificant cancers, raising concerns about overdetection.

Microcalcifications (MCs), which are small calcium deposits, are common mammographic findings where they typically appear as high optical density structures. Nearly 50% of the biopsied MCs are associated with ductal carcinoma in situ (DCIS) [5], an early form of cancer but a nonobligate precursor to invasive cancer [6, 7]. MCs are reported by radiologists using a set of qualitative descriptors related to morphology (shape) and distribution, as defined by the American College of Radiology Breast Imaging Reporting and Data System (BI-RADS) using a combination of full-field digital mammograms (FFDMs) and magnification views. Descriptors correspond to varying levels of suspicion for cancer. For example, amorphous MCs are assigned a moderate suspicion level (i.e., BI-RADS 4B) with a positive predictive value (PPV_3_)^1^ of 21% [8]. However, the assigned level of suspicion can be open to interpretation and varies by radiologists due to subtle differences in MCs’ size, shape, texture, and heterogeneity in the background tissue [9]. Hence, determining whether a group of calcifications is associated with malignancy is challenging, and the current PPV_3_ of biopsied suspicious MCs on 2D mammography is in the range of 20–41% [10].

Many computerized methods have been developed to aid radiologists in detecting MCs [9, 11–15]. These methods, generally categorized as computer-aided detection (CADe) systems, automatically mark groups of suspicious MCs in mammograms. Current CADe systems achieve high sensitivity but at the cost of a large number of false positive marks per mammogram, increasing the interpretation time. In work similar to ours, Wang et al. [9] developed a context-sensitive deep convolutional neural network, focusing on detecting MCs with low false positives. Their approach generated candidate locations using a difference of Gaussians (DoG) blob detection, filtering out non-MCs using a convolutional neural network. Our approach goes beyond detection by segmenting the boundaries of each MC.

In addition, studies have shown that using shape and intensity features from segmented MCs can improve malignancy classification [16–20]. Precise segmentation also allows for more accurate quantitative characterization of the shape and distribution of MCs and texture analysis of the surrounding breast parenchyma that could be used to classify cancerous regions better. Prior studied techniques include wavelet transform for isolating high-frequency components [21, 22], gray-level morphological operations [11, 23–26], fuzzy logic [27], and binary pixel classification using machine learning [17]. Although segmentation of MCs is performed in these studies, all but Ciecholewski [24] evaluated the performance of their algorithm as a detection task (using free-response operating characteristic analysis), not a segmentation task (measured by the overlap of delineated regions). Ciecholewski reported an intersection over the union of 70.8% between the segmented MCs and the radiologist annotations on a set of 200 regions, which we use as a basis for comparing our algorithm.

In this study, a quantitative morphology-based approach for characterizing MCs is demonstrated. Given a 2D digital mammogram, we initially identify bright salient structures using the DoG blob detection algorithm. Hessian analysis is then applied to segment these structures. Next, dense regression is employed to segment regions containing structures that are likely to be MCs. Dense regression has been used for similar tasks such as cell and nuclei detection [28, 29], retinal optical disc and fovea detection [30], and focal vascular lesion localization on brain MRI [31]. The idea is that human experts’ reference annotations are mapped to a smooth proximity function that reaches its maximum value when corresponding to the annotated points. Dense regression models are then trained to map the input mammogram to the proximity function. The proximity function method is advantageous when objects are annotated by a single pixel rather than their actual boundaries (e.g., many MCs are tiny and time-consuming to delineate). A fully convolutional network with pretrained weights is utilized to perform dense regression. The outputs of the dense regression model and the blob segmentation algorithm are combined to generate the final MC segmentation.

The contributions of our work are summarized as follows:

- Obtaining precise annotations of all MCs is impractical, given the time and labor required. As a result, manual annotations of MC boundaries are often inconsistently drawn with high variability. To accommodate this uncertainty, we use proximity functions to represent individual MCs as part of regression model training.
- A dense regression model and a novel blob segmentation algorithm are applied to generate MCs’ accurate segmentation while achieving fewer false positives than comparable state-of-the-art algorithms.
- Our approach is trained and tested on a set of screening and diagnostic mammograms from two cohorts (INbreast and local data). We demonstrate the generalizability of our approach by applying our method to a set of magnification views.

## Materials and Methods

### Data

#### INbreast Dataset

For model training and internal validation, we utilized a public dataset called INbreast [32], a collection of 2D screening and diagnostic FFDMs, which were generated using a Siemens MammoNovation system. 115 screening cases with 410 images were collected at a 0.070 mm per pixel resolution and 14-bit greyscale. The dataset included detailed annotations provided by two experts for several types of lesions (i.e., masses, MCs, asymmetries, and distortions). Fifty-six cases had pathology-confirmed diagnoses, of which 45 were cancerous (DCIS and invasive). We used 294 images (147 craniocaudal (CC) and 147 mediolateral oblique (MLO) views) from 86 screening cases with annotations of individual MCs. MCs were annotated in two ways: (1) small MCs were annotated by a single pixel to denote their location, and (2) larger MCs were annotated using pixel-wise contours. It should be noted that a guideline of what was considered small versus larger MC was not reported.

#### Local Dataset

As an additional test set, data collected retrospectively from patients who had 2D diagnostic FFDMs performed at our institution, following an institutional review board (IRB)-approved protocol, was used. The dataset consisted of 79 diagnostic cases with 141 FFDM images (46 CC, 21 MLO, and 74 mediolateral (ML) views) where MCs were present. All images were acquired using Hologic Selenia full-field digital mammography equipment at a 0.070 mm per pixel resolution and 12-bit greyscale. After collecting the data, suspicious MCs were annotated by a breast fellowship-trained, board-certified radiologist with five years of experience. An open-source medical image viewer, Horos, was utilized to generate the annotations. Individual MCs were annotated by single pixels indicating their locations. A second board-certified radiologist annotated a sample of 5 cases to assess the annotation task’s interreader reliability. The index of specific agreement and the kappa statistic was determined. The two radiologists’ agreement was moderate, with an index of specific agreement of 0.664 (0.606–0.729, 95% confidence interval), see Supplementary Information.

#### Local Magnification View Dataset

We used magnification views obtained from 248 patients with amorphous calcifications seen at our institution to evaluate the performance of a malignancy classification. The model utilized features extracted from segmentations generated by our approach to classifying cases as benign or malignant (see the case study described in the Sect. “Case study: Identifying breast cancers among amorphous calcifications’’).

### Overall Approach

**Fig. 1:**
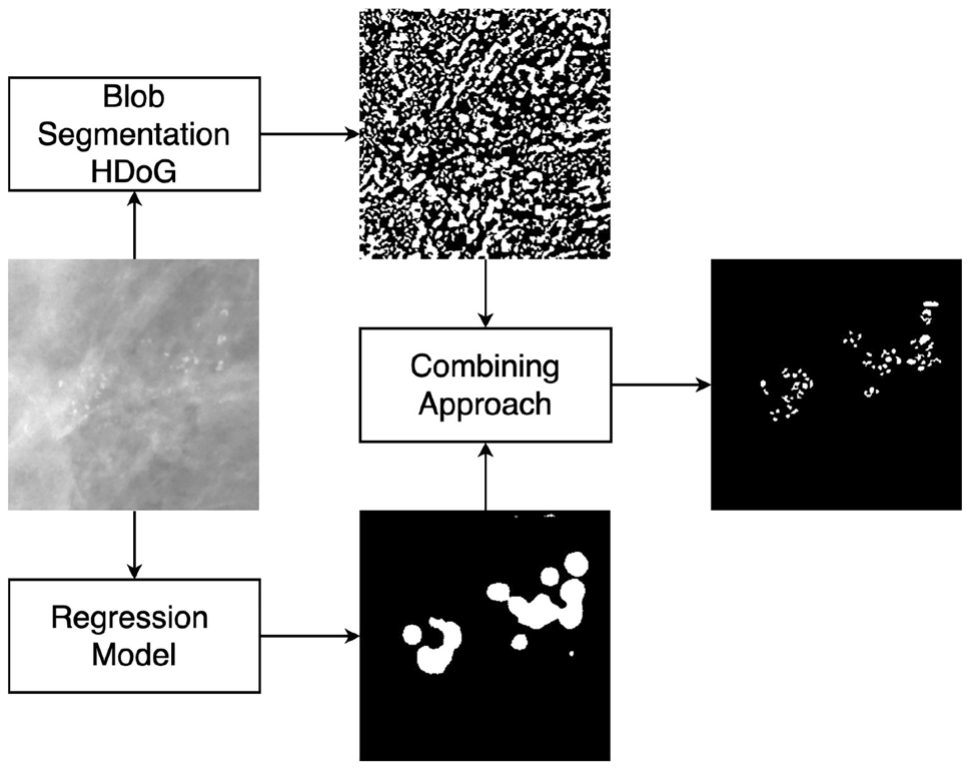
Approach for segmenting MCs. While the segmentation is performed on the entire 2D FFDM, for visualization purposes, a small patch is shown. In the upper branch, blob segmentation is performed to segment bright blob-like and tubular structures. In the lower branch, a regression convolutional neural network gives a continuous function with a higher response close to MCs. A threshold is then applied to segment regions where MCs are likely to be present. The two branches’ output is combined based on an overlap criterion (e.g., retain blobs that have at least 30% overlap with the segmented region), resulting in the final segmentation mask

The overall approach is illustrated in Fig. 1.

### Blob Segmentation

The first stage is the segmentation of granular structures that are candidate MCs. To generate candidate MC segments, we developed Hessian DoG for blob segmentation. This module’s objective is the accurate segmentation of bright salient structures that are candidate MC objects, as shown in Fig. 1.

Scale-space theory is a framework formulated to represent signals at multiple scales. The Gaussian scale-space representation of an image *I*(*x*, *y*) is defined as [33]:

1 $$\begin{aligned} \begin{aligned} L(x, y; \sigma ) = G(x, y; \sigma ) *I(x, y), \end{aligned} \end{aligned}$$

where $*$ is the convolution and $G(x,y,\sigma )$ the two-dimensional Gaussian function

2 $$\begin{aligned} \begin{aligned} G(x,y; \sigma )&= \frac{1}{2\pi \sigma ^2} e^{-(x^2+y^2)/(2\sigma ^2)}. \end{aligned} \end{aligned}$$

In the DoG method, blobs with associated scale levels are detected from scale-space maxima of the scale-normalized DoG function. The normalized DoG function is defined as:

3 $$\begin{aligned} \begin{aligned} DoG(x,y;\sigma )&= \frac{\sigma }{\Delta \sigma } \left( L(x,y;\sigma +\Delta \sigma )-L(x,y;\sigma ) \right) \end{aligned} \end{aligned}$$

where $\Delta \sigma$ is the difference between two scales. To construct the DoG scale-space representation, a sequence of scales is considered $\sigma _n = k^n \sigma _{\min }$ where *k* is a constant multiplicative factor and $n=[0,1,\cdots , n_{\max } ]$. The DoG representations Eq. (3) are computed for all adjacent scales (i.e., $\Delta \sigma = \sigma _{n+1}-\sigma _n$) forming a 3-dimensional representation:

4 $$\begin{aligned} \begin{aligned} DoG(x,y,n)&= \frac{\sigma _n}{\sigma _{n+1}-\sigma _n} \left( L(x,y;\sigma _{n+1})-L(x,y;\sigma _n) \right) \end{aligned} \end{aligned}$$

with *x*, *y* the two spatial dimensions and $n=[0,1,\cdots , n_{\max -1} ]$ a scale dimension. Local maxima in the 3-dimensional representation are computed giving a blob set $(x^{(i)},y^{(i)},\sigma ^{(i)})$ where *i* identifies each blob. The number of blob detections is controlled by a threshold, $T_\mathrm {DoG}$, that is applied as a lower bound on the DoG representation before obtaining the local maxima. Moreover, in the case of overlapping blobs, the smaller blob is eliminated if the overlapping fraction is greater than the threshold $O_\mathrm {DoG}$.

The DoG algorithm outputs the location and scale of the detected blobs. To extract the blob shapes, we extended this method using Hessian analysis. The geometrical structure (i.e., convexity structure) of a blob-like object can be described by the eigenvalues of the Hessian [34]. In particular, a bright blob-like structure corresponds to two negative and large eigenvalues, whereas a bright tubular structure corresponds to one large negative eigenvalue and a small eigenvalue of an arbitrary sign. These structures correspond to the target MC candidates.

The Hessian DoG (H) representation at scale $\sigma$ is given by

5 $$\begin{aligned} \begin{aligned} \mathrm {H}(x,y;\sigma ) = \begin{pmatrix} \frac{\partial ^2 DoG(x,y;\sigma )}{\partial x^2} &{} \frac{\partial ^2 DoG(x,y;\sigma )}{\partial x \partial y}\\ \frac{\partial ^2 DoG(x,y;\sigma )}{\partial x \partial y} &{} \frac{\partial ^2 DoG(x,y;\sigma )}{\partial y^2} \end{pmatrix}. \end{aligned} \end{aligned}$$

*H* is computed across all scales in the sequence $\sigma _n$. At each scale, the following constraints are imposed:

6 $$\begin{aligned} \begin{aligned} \mathrm {tr}(H)< 0 \wedge \left( \det (H)<0\quad \vee \quad \frac{\det (H)}{\mathrm {tr}(H)^2} \le h_{\mathrm {thr}} \right) \end{aligned} \end{aligned}$$

where $h_{\mathrm {thr}}$ is a tunable parameter. The constraints ensure that the Hessian is either negative definite or has a small positive eigenvalue. In this way, only bright salient blob-like and tubular structures are segmented. The constraint generates a binary mask at each scale. Iterating over the blob set found in the DoG algorithm $(x^{(i)},y^{(i)},\sigma ^{(i)})$, the corresponding objects are found in the Hessian masks. More specifically, for the Hessian mask at scale $\sigma ^{(i)}$, the object spanning the location $(x^{(i)},y^{(i)})$ is found. The output of this step consists of all detected objects merged into a single binary mask.

### Regression Convolutional Neural Network

While the blob segmentation step identifies objects that are candidate MCs, many will be false positives. This step identifies regions where MCs are most likely present by segmenting the MCs’ area to choose relevant MC objects from the previous stage. This task was performed using a fully convolutional neural network, commonly used in image segmentation, as the regression model. The model’s output is a smooth proximity map reaching a maximum value at the predicted MC locations.

The MC region segmentation task is analogous to cell and nuclei detection in microscopy images. The two tasks share the following characteristics: (1) they are highly imbalanced (i.e., the positive class captures a small region compared to the background within an image, and it often consists of many small structures), (2) the background is highly inhomogeneous, (3) individual objects exhibit large variation in sizes, shapes, and textures, (4) boundaries of the structures are often blurry, (5) the resolution of both types of images is large, and (6) the annotations are usually a mixture of individual points or exact boundaries. Inspired by this analogy, we adapted methods previously used in cell and nuclei detection [28, 29]. In [28], the authors used regression to detect cell centers. The human-annotated binary masks containing cell centers’ locations were transformed into a continuous function flat on the background with localized peaks at each cell’s center. These functions were then used to train a Random Forest Regression algorithm on a set of image patches. The cell centers were identified with local maxima in the model’s output. In [29], the authors showed that the same technique could be applied using a deep learning model. Their regression model was a fully convolutional neural network with a large receptive field capable of encoding high-resolution information.

**Fig. 2:**
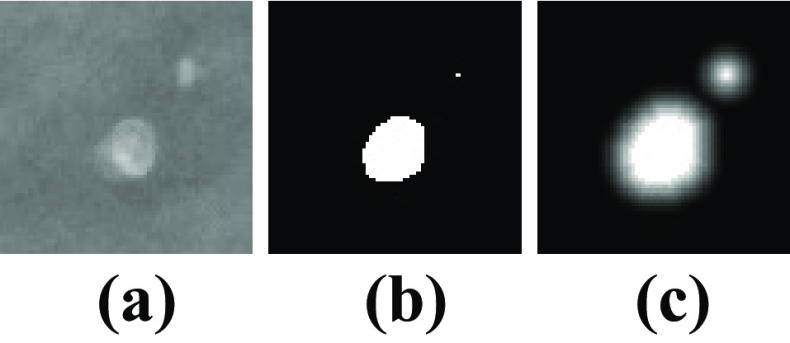
**a** A mammographic image patch which includes MCs; **b** The corresponding annotation mask; **c** The corresponding proximity function map with parameters $\xi =10$ and $\alpha =1$

Our MC segmentation model is formulated as follows: Given a mask generated from reference annotations $M(x,y) \in \{0,1\}$, the MC locations are given by $\{(x_i, y_i)\}$ where $M(x_i,y_i) = 1$. The proximity function is then defined as:

7 $$\begin{aligned} P(x,y) = \max _i g(x,y,x_i,y_i) \end{aligned}$$

8 $$\begin{aligned} \begin{aligned} g(x,y,x_i,y_i)&= {\left\{ \begin{array}{ll} (e^{\alpha (1-r/\xi )}-1)/(e^\alpha -1), &{} r \le \xi \\ 0, &{} r > \xi \end{array}\right. } \end{aligned} \end{aligned}$$

9 $$\begin{aligned} r = \sqrt{(x-x_i)^2+(y-y_i)^2} \end{aligned}$$

where $\alpha , \xi$ are tunable parameters. The function maps MC locations on an exponentially curved surface, expanding to a distance $\xi$ with decay rate $\alpha$ before it vanishes. An example of the transformation is illustrated in Fig. 2. This transformed mask compensated for the fact that we had mixed quality annotations (i.e., point-like and exact) and forced the model to learn information from the precise locations of MCs and the surrounding background.

We constructed a model which predicts the proximity function *P*(*x*, *y*) given the image *I*(*x*, *y*). A feature pyramid network (FPN) [35] was used with Inception-v4 [36] as the backbone. The FPN architecture was introduced for applications such as region proposal, object detection, and instance segmentation. It adopts a pyramidal shape structure similar to many segmentation networks, such as the U-net [37], with an encoder that produces semantic features at different scales and a decoder that combines the encoder features by upsampling them.

**Fig. 3:**
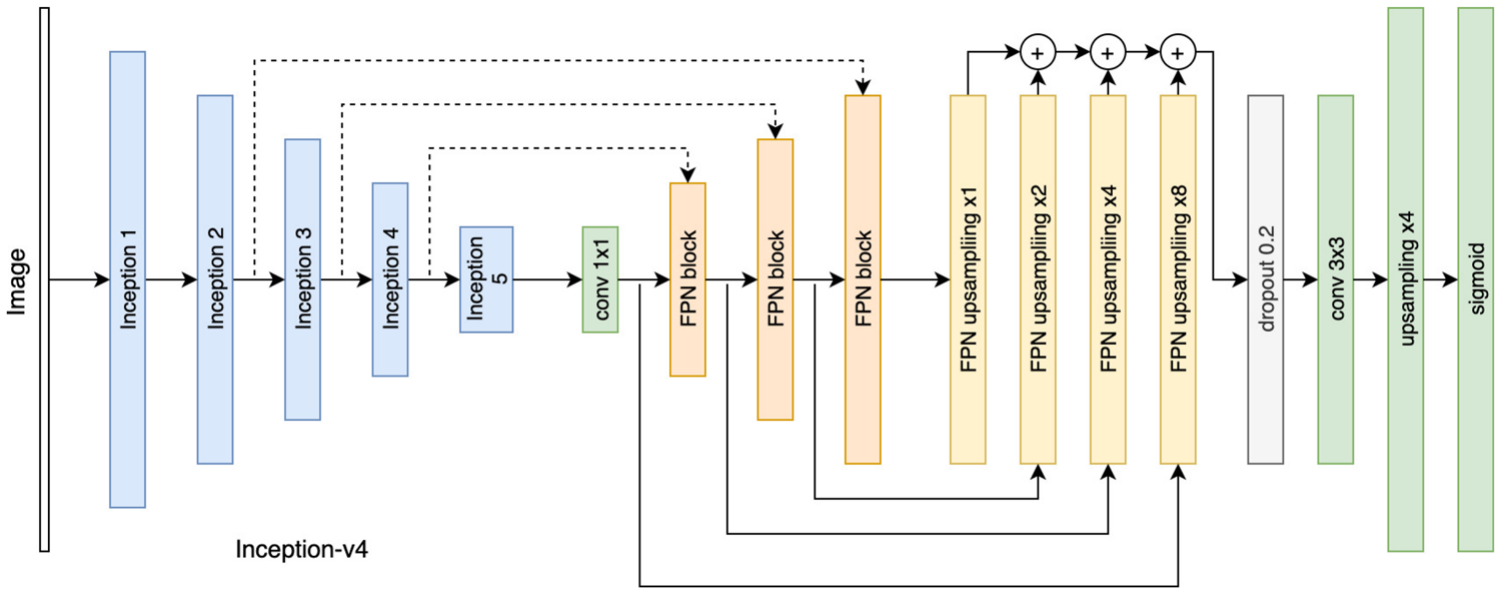
FPN with Inception-v4 encoder used for regression. In the encoding branch, the image is processed with the Inception-v4 classification network. Skip connections (dashed lines) are inserted after layers where the output was reduced in spatial size by factors of 4, 8, 16, and 32, respectively. The skip connections feed FPN blocks where they undergo a series of convolutions. The outputs are upsampled independently by factors of 1, 2, 4, and 8, respectively. Their outputs are added and inserted in a spatial dropout layer activated only during training for regularization purposes. After dropout, convolutions are followed by an upsampling by a factor of 4 to match the original image size and the sigmoid activation function

The FPN architecture is suitable because it allows features from all scales to independently contribute to the final prediction. The network is illustrated in Fig. 3. The network consists of an encoding and a decoding branch. In the encoding branch, the Inception-v4 architecture was adopted with weights pretrained on ImageNet [38]. Features were extracted at four scales (down-sampled compared to the original image by factors of 4, 8, 16, and 32). The features were then transferred to the decoding branch via skip connections. To match their spatial sizes, they were upsampled by factors of 1, 2, 4, and 8. The resulting features were aggregated using addition and further upsampled to match the image size. The number of output channels was set to 1 and passed through a sigmoid function to generate a value between 0 and 1. This value was thresholded to achieve the final segmentation.

The model was trained using a soft Dice loss function, which was introduced as an optimization objective in biomedical segmentation applications [39, 40]. The formulation in [39] was used:

10 $$\begin{aligned} \begin{aligned} L_{\mathrm {DICE}}(\hat{P}, P) = 1 - \frac{2 \sum \limits _{x,y} P(x,y) \hat{P}(x,y) +\epsilon }{\sum \limits _{x,y} (P(x,y)+\hat{P}(x,y)) +\epsilon } \end{aligned} \end{aligned}$$

with $\epsilon$ set to 1 where $\epsilon$ was introduced for numerical stability and *P* and $\hat{P}$ correspond to the target and predicted proximity map, respectively. A segmentation binary mask was generated by applying a cut-off on the resulting proximity mask, $\hat{P}(x,y) \ge p_{thr}$ where $p_{thr} \in [0,1]$.

The regression model was trained using patches extracted from the images and corresponding masks. We applied the sliding window approach with a patch size of 512 pixels and a stride of 480 to permit overlapping patches. Only patches with annotated MCs present were considered. From INbreast cases, 1045 patches were extracted from the training set and 329 from the validation set. From our local dataset, 252 patches from the training set were extracted.

The mask patches were transformed using the proximity function map Eq. (7). We set $\xi = \{ 6,8,10,12\}$ for the characteristic distance and $\alpha = \{ -1,-2, 10^{-4}, 1,2\}$ for the decay rate. The proximity function Eq. (7) is not well defined when $\alpha =0$.

Data augmentation was performed to enrich the training set by randomly applying horizontal flipping, magnification, spatial translations in both directions, cropping, contrast enhancement, brightness adjustment, and gamma correction (details are given in supplementary materials). The resulting patches are 320x320 pixels in size. The soft Dice loss was used to compute the error between target and predicted proximity functions Eq. (10). The model was trained for 40 epochs using the adaptive moment estimation (Adam) optimization method [41] with mini-batch size 8, learning rate $10^{-4}$, $\beta _1 = 0.9$, $\beta _2 = 0.999$ and $\epsilon =10^{-8}$. At the end of each epoch, the model was evaluated on the image patches of the INbreast validation set, with the average IoU per patch as the metric. The model achieving the highest IoU over all epochs was kept. The Inception-v4 weights were initialized with weights pretrained on ImageNet. The rest of the model weights were initialized randomly following He initialization [42]. The configuration $\xi = 10$, $\alpha =1$ achieved the highest performance for our approach on the validation set. We updated our model to work with local data by training the model with an additional 40 epochs using a combination of patches from the INbreast and local datasets.

### Output Generation

Blob segmentation detects bright objects, whereas the regression CNN outputs a mask of relevant MC regions. The intersection of these outputs results in the final set of detected MCs and their segmentations. We retained the Hessian DoG objects that overlap $> o_{\mathrm {thr}}$ with the CNN region mask, where $o_{\mathrm {thr}}$ is a tunable parameter, representing percentage overlap.

### Comparison Methods

We compared our approach against two state-of-the-art methods. For the MC detection task, our approach was compared to the paper by Wang and Yang [9], which used two subnetworks, one focusing on the local features and the other on features extracted from the background tissue around the location. They reported a detection performance of 80% true positive fraction (TPF) at a false positive rate of 1.03 FPs/cm^2^. We implemented their context-sensitive deep neural network, classifying a location as MC or non-MC, training our implementation on the INbreast dataset. We implemented DoG based on their reported parameters adjusting the scales to the resolution of our dataset.

For the MC segmentation task, our approach was compared to Ciecholewski [24], where MC segmentation was performed using morphological operations. In the first step, morphological operators were applied to the original image to detect the MCs’ locations. Specifically, a morphological pyramid was generated using the closing-opening filter. Differences in the pyramid representations of the original image were obtained and combined using the extended maximum of the original image and morphological reconstruction. In the second step, the MC shapes were extracted using watershed segmentation, where the output of the first step was utilized as a marker.

### Evaluation Metrics

The segmentation performance was assessed using Intersection over the Union (IoU). We defined IoU per object as the averaged IoU between each reference annotation object and the object with the most overlap within the prediction mask^2^. The mean IoU between the background and the positive MC class per image was computed to evaluate the image-wise segmentation. The IoU per MC object was measured to examine the performance of segmenting individual MCs.

The detection performance of our approach was evaluated using Free-Response Operating Characteristic (FROC) analysis, similar to prior work [9, 12]. In FROC analysis, the true positive rate (TPR) was plotted against false positive detections per image unit area (cm^2^). The analysis required the definition of localization rules to determine true positives. We defined a detected object as a true positive if its distance from a ground truth object was at most 5 pixels (0.35 mm)^3^ or if it demonstrated an IoU value of at least 0.3 with a ground truth object.

## Results

### Training, Validation, and Test Sets

The INbreast dataset was partitioned into a training set with 51 cases (173 images), a validation set with 17 cases (56 images), and a test set with 18 cases (65 images). The local dataset was partitioned into a training set for fine-tuning the model with 112 images and a held-out test set with 29 images. We used the INbreast validation set to fine-tune our approach and the INbreast test set to assess the performance. Cases were kept independent (i.e., all images from an individual case were included within the same subset) to avoid potential bias.

### Model Selection and Optimization

We evaluated our model across hyperparameters using the INbreast validation set. The FROC analysis is presented in Table 1, and the mean IoU per image and IoU per object are summarized in Table 2. For the FROC analysis, 100 bootstrap samples were used to find the partial area under the curve (pAUC) in each experiment. The pAUC was computed for the range between 0 and 1 FPs per unit area, and the 95% confidence interval was reported. For the computation of the segmentation metrics, a threshold on the predicted proximity function was applied. To determine the optimal threshold for each experiment, we referred to the corresponding FROC curve and found the point closest to a TPR of 1 and a false positive per unit area of 0. All configurations performed similarly in terms of the FROC analysis and segmentation metrics. We chose the model with the highest mean value of the FROC pAUC with $\xi =10$ and $\alpha =1$. We set $\sigma _{\min } = 1.18$, $\sigma _{\max }=3.1$, overlapping fraction $O_\mathrm {DoG}=1$, DoG threshold $T_\mathrm {DoG}=0.006$ and Hessian threshold $h_\mathrm { thr}=1.4$. To optimize the final output, we examined $o_\mathrm {thr} = \{0.2, 0.3, 0.4, 0.5, 0.6\}$ to determine the overlap threshold. Setting $o_\mathrm {thr}=0.3$ achieved the highest performance on the validation set.

**Fig. 4:**
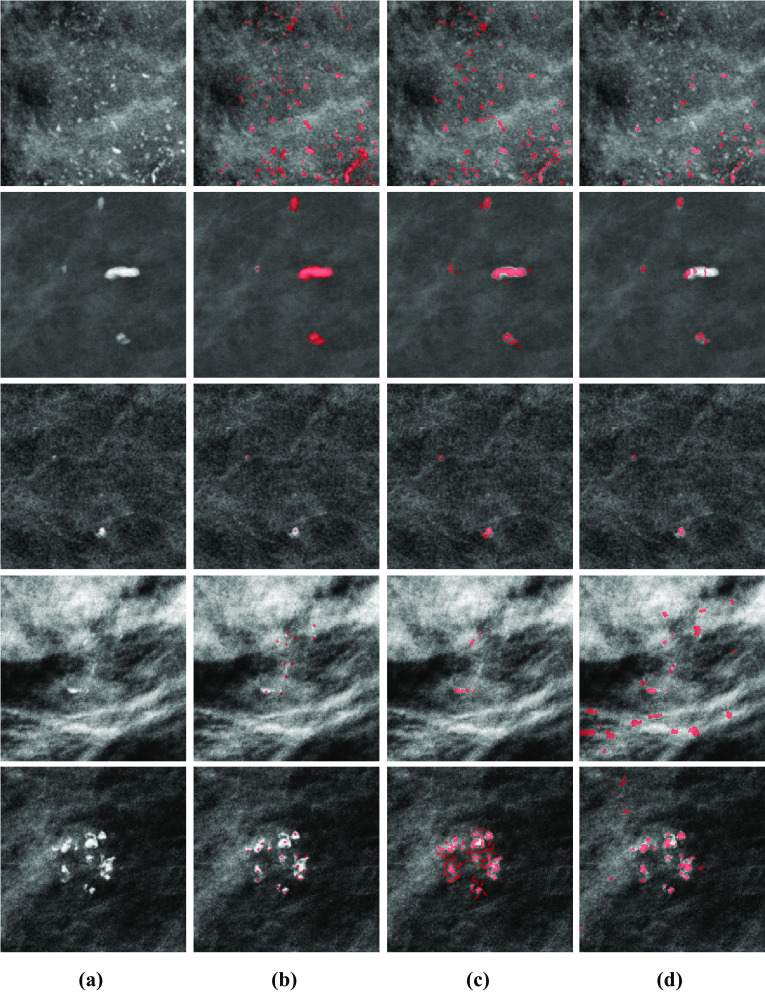
Five 256x256 patches extracted from different mammograms showing the results of our approach and a comparison method on a variety of microcalcifications. From left to right: **a** unannotated images, **b** reference annotations, **c** results using our approach, and **d** results applying the approach described in [24]. The first three rows are from INbreast data, and the last two are from local data. For better visualization, the patches were normalized. Note the inherent difference in the appearance of the mammograms between INbreast and local data due to differences in acquisition systems

**Table 1:** Detection performance for different regression models on the validation set. The highest pAUC is bolded

$\alpha /\xi$	6	8	10	12
-2	0.804 ± 0.048	0.812 ± 0.049	0.793 ± 0.040	0.773 ± 0.045
-1	0.783 ± 0.058	0.808 ± 0.047	0.790 ± 0.047	0.794 ± 0.045
10^-4^	0.813 ± 0.054	0.783 ± 0.056	0.790 ± 0.044	0.784 ± 0.048
1	0.799 ± 0.054	0.776 ± 0.056	**0.819 ± 0.046**	0.790 ± 0.053
2	0.775 ± 0.056	0.791 ± 0.055	0.802 ± 0.049	0.789 ± 0.053

**Table 2:** Segmentation results of different regression models on the validation set. The highest IoUs are bolded

Mean IoU per image				
$\alpha /\xi$	6	8	10	12
-2	0.593 ± 0.109	0.590 ± 0.102	0.576 ± 0.088	0.560 ± 0.066
-1	0.590 ± 0.105	0.579 ± 0.094	0.572 ± 0.089	0.566 ± 0.077
10^-4^	0.596 ± 0.115	0.584 ± 0.098	0.586 ± 0.097	0.584 ± 0.093
1	**0.619 ± 0.125**	0.601 ± 0.109	0.583 ± 0.105	0.587 ± 0.098
2	0.610 ± 0.123	0.588 ± 0.108	0.592 ± 0.105	0.580 ± 0.094

IoU per image				
$\alpha /\xi$	6	8	10	12
-2	0.645 ± 0.207	0.645 ± 0.206	0.648 ± 0.200	0.626 ± 0.228
-1	0.647 ± 0.203	0.648 ± 0.201	0.643 ± 0.208	0.640 ± 0.212
10^-4^	0.648 ± 0.201	0.644 ± 0.207	0.641 ± 0.214	0.644 ± 0.207
1	0.649 ± 0.200	0.646 ± 0.206	0.647 ± 0.203	0.643 ± 0.208
2	**0.650 ± 0.197**	0.648 ± 0.202	0.649 ± 0.200	0.643 ± 0.208

### Detection and Segmentation Results

**Fig. 5:**
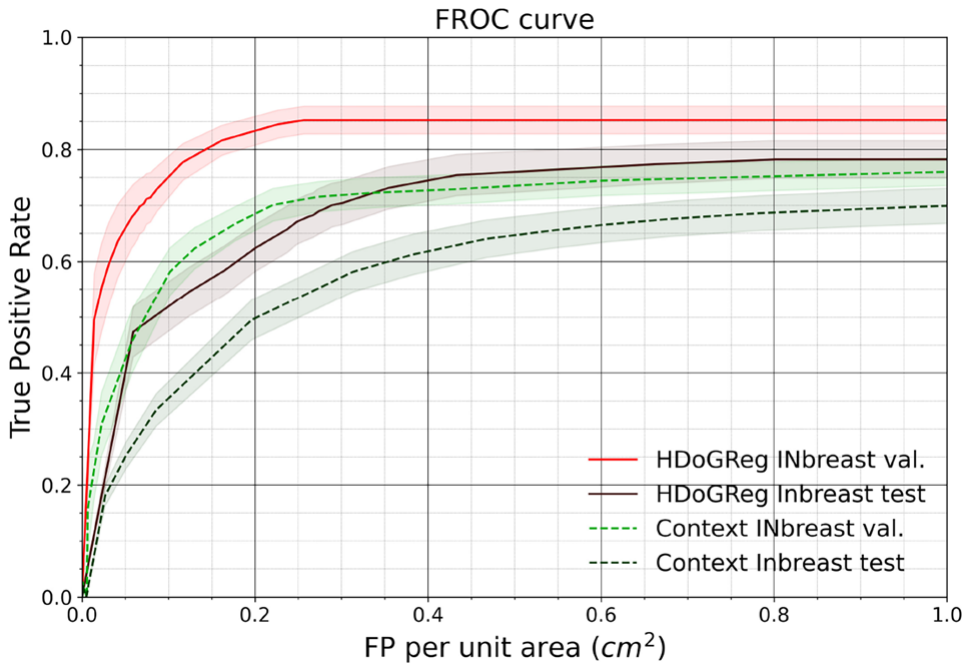
Individual MC FROC analysis for our final model compared with a baseline model

Figure 5 compares the detection performance between our method and the method of Wang and Yang [9]. The true positive detection rate on the y-axis was plotted against the false positive counts per unit area (1 $cm^2$). The FROC analysis was performed on the INbreast validation and test sets. Our method achieved FROC pAUC 0.819 ± 0.046 with a TPR of 0.852 at 0.4 false positives per unit area on the validation set. On the test set, the FROC pAUC was 0.697 ± 0.078 with a TPR of 0.744 at 0.4 false positives per unit area. In comparison, our implementation of [9] achieved FROC pAUC 0.703 ± 0.057 and 0.581 ± 0.072 in the validation and test sets, respectively.

**Fig. 6:**
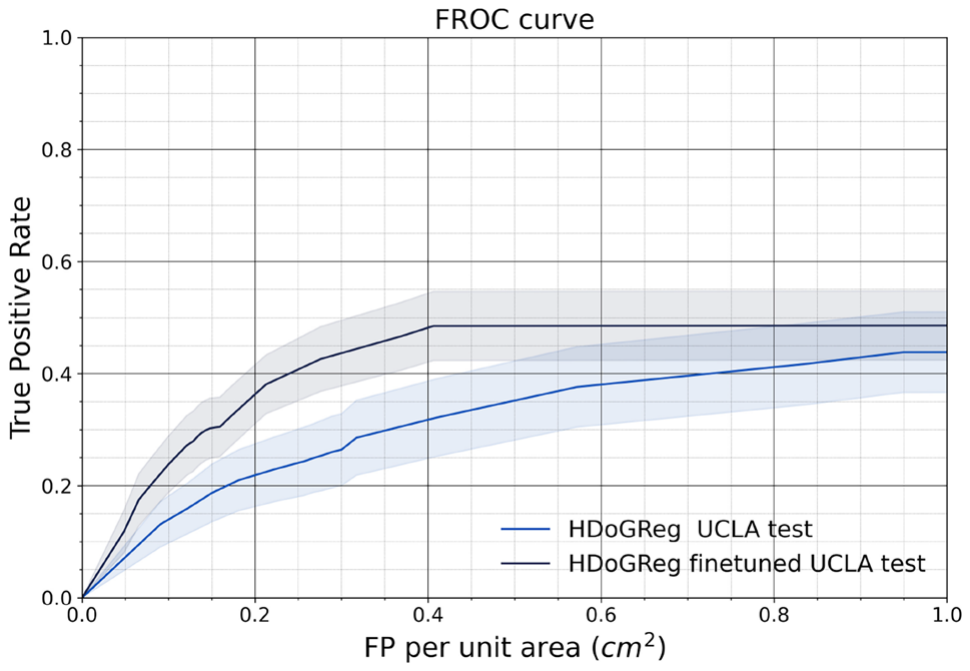
Individual MC FROC analysis on local data

Figure 6 shows the detection performance of our approach on the local dataset. We also compared the model’s performance trained solely on INbreast data and fine-tuned on local data. The performance of the two models was comparable since the original model achieved 0.313 ± 0.109 FROC pAUC, and the fine-tuned model achieved 0.420 ± 0.107. However, for the range of 0.2 to 0.6 FPs per unit area, the fine-tuned model outperformed the original based on TPR.

Table 3 reports the segmentation performance of our approach on the INbreast validation and test sets. For comparison, the segmentation results of the morphological method of Ciecholewski (see the Sect. “Comparison Methods’’ ) are also presented. Based on the paired Wilcoxon signed-rank test, we achieved superior performance in both mIoU per image and IoU per object for both subsets with $p<0.01$. Figure 4 presents a sampling of model outputs.

**Table 3:** Segmentation Results of Final Model on Validation and Test Sets

Our Approach		
Metric/dataset	Validation	Test
mean IoU per image	0.583 ± 0.105	0.670 ± 0.121
IoU per object	0.647 ± 0.203	0.607 ± 0.250
Ciecholewski et al. [24]		
mean IoU per image	0.517 ± 0.037	0.524 ± 0.034
IoU per object	0.408 ± 0.286	0.363 ± 0.278

### Case Study: Identifying Breast Cancers Among Amorphous Calcifications

We present a case study that utilized features computed from regions segmented by our method to classify whether MCs identified as amorphous were benign or malignant. We compared the predictive value of these features with those computed from regions delineated by another “baseline” method [24].

#### Data

The local magnification view dataset consisted of diagnostic exams performed at our institution between 2017 and 2019. In particular, 284 mammographic cases with biopsied amorphous MCs were selected. The cases were chosen such that for the same case and laterality (left/right breast), all biopsy results were either benign or malignant (high-risk results were omitted). The cases corresponded to a total of 642 diagnostic images (318 ML/MLO/LM and 324 CC). A doctoral-trained researcher annotated the most suspicious regions, providing the regions of interest (ROIs) as bounding boxes. A board-certified radiologist validated these ROIs. In total, 674 ROIs were provided, i.e., 612 images with one ROI, 28 images with two ROIs, and 2 images with three ROIs. Incorporating the pathology information, 390 (57.9%) ROIs were benign and 284 (42.1%) malignant. To eliminate the annotation bias associated with the size and shape of ROIs, we transformed the bounding boxes of ROIs to a fixed size. The mean ROI height and width were 222 and 256 pixels, respectively. Therefore, we decided to transform each bounding box to a 256x256 pixel size retaining its center location. Another reason for adopting fixed-sized ROIs was to make the classification model focus on segmentation-related features.

#### MC Segmentation

MCs were segmented using two different methods:

1. Our Approach: For the regression network, the FPN was trained on both local and INbreast data (see the Sect. “Data’’). For the Hessian DoG blob segmentation, we fine-tuned the parameters mentioned in Sect. “Blob Segmentation’’ to achieve the best classification performance.
2. We used the method developed by Ciecholewski [24] as the “baseline” segmentation method, described in the Sect. “Comparison Methods’’.

#### Feature Extraction

Upon segmentation of the ROIs, relevant features were extracted (n=31). The extracted features are categorized into two main groups: (1) regional features describing all ROI MCs as a whole and (2) individual MC features. The regional features were: the area of the foreground (all MCs), the area of the convex hull enclosing all MCs, major and minor axis length, the orientation of the major axis with respect to the horizontal line, eccentricity, solidity, moments of inertia (n=2), Hu moments (n=7) and number of MCs. The MCs were also described individually by their area, major and minor axis length, maximum, minimum, and mean intensity within, and eccentricity. Individual MC features were statistically aggregated using mean and standard deviation per ROI.

#### Classification

Features were inputted into a gradient tree boosting classifier. Gradient boosting generates weak predictive models, i.e., in our case, decision trees, which are enhanced in each iteration, targeting residual errors, and are linearly combined to give the final model. We applied fivefold cross-validation to train and test our task. Partitioning the data into folds was performed patient-wise. For each training and test split, the data were pre-processed using imputation by mean value followed by standardization (i.e., subtracting the mean and scaling to unit variance). Imputation was needed for cases where the segmentation was empty. The parameters for both imputation and standardization were derived from the training set and applied to both training and test sets.

**Fig. 7:**
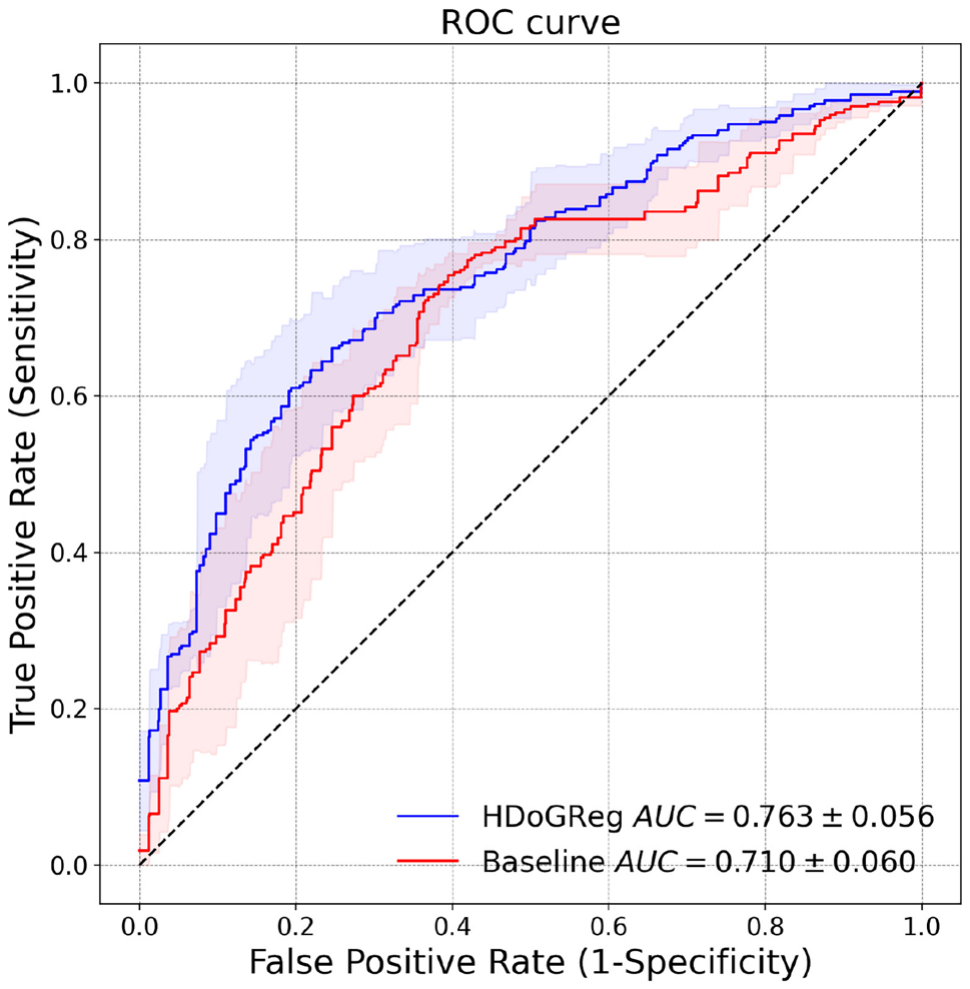
ROC curves for the classification models obtained using our approach and a baseline segmentation method. Lines correspond to mean values across folds and the filled area captures one standard deviation

**Fig. 8:**
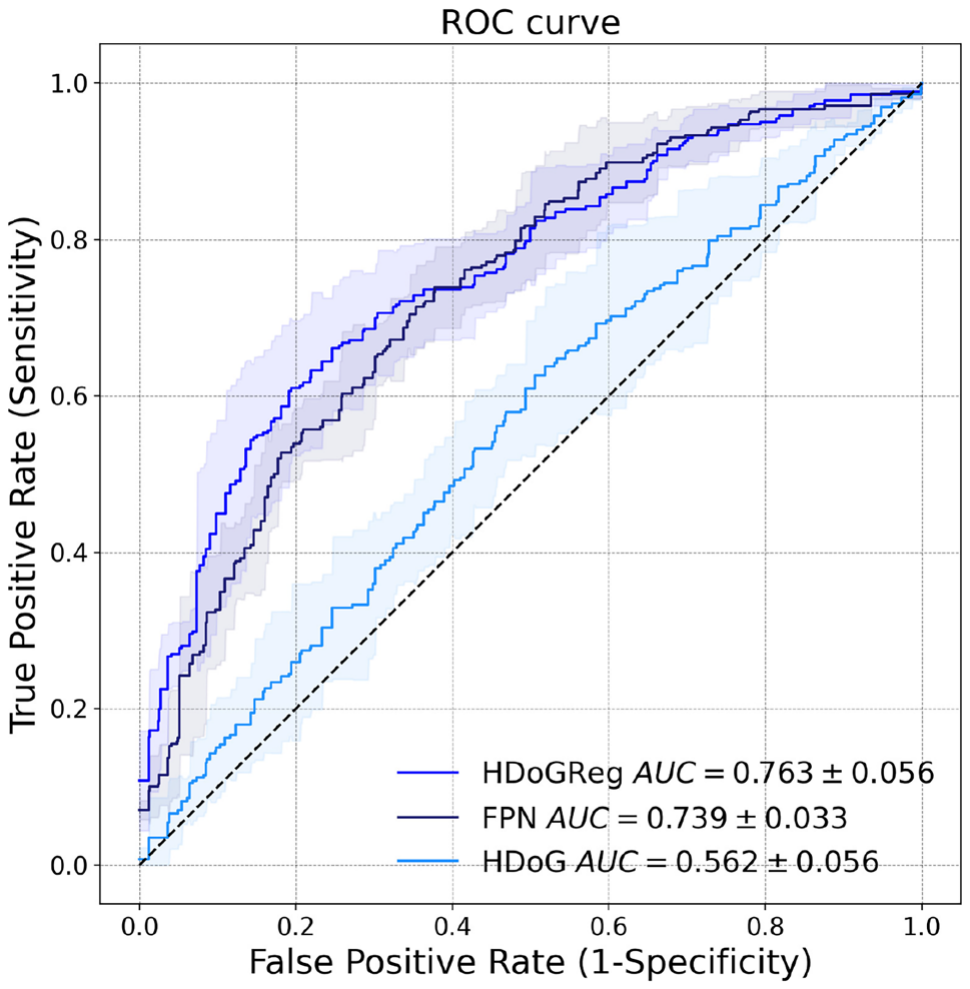
ROC curves breaking down our method’s segmentation performance in terms of its constituent parts. The ROC curves for blob segmentation and the convolutional regression model (FPN) are presented. Lines correspond to mean values across folds and the filled area captures one standard deviation. The graph highlights the complementary value the two components (Hessian DoG and FPN) contribute to the overall method

#### Evaluation

Classification was performed on different sets of features derived from different MC segmentation techniques. ROC analysis was performed to evaluate each model, and classification metrics were obtained, i.e., ROC AUC, accuracy, sensitivity, specificity, and positive predictive value. The mean and standard deviation of each metric was computed across folds.

The parameters of each segmentation method were fine-tuned, and the configurations that achieved the highest ROC AUC were kept (details are given in Supplementary Information). The best models obtained using our method and baseline segmentations were compared using ROC analysis. Figure 7 depicts the ROC curves of each model. Other classification metrics are presented in Table 4. We deliberately chose operating thresholds to achieve high sensitivity (close to 90%) for both models. The model trained on features generated from our method’s segmentations achieved superior values for all other classification metrics.

In Fig. 8, we compared the performance of our approach with respect to its components: FPN and Hessian DoG blob segmentation. Although the performance of blob segmentation was low (AUC=0.562 ± 0.056), the combination of blob segmentation and FPN resulted in the highest performance (AUC=0.763 ± 0.056).

**Table 4:** Fivefold cross-validation classification metrics compared between our method and baseline segmentations. Mean values and standard deviations across folds are presented. The threshold is chosen to achieve a sensitivity closest to 0.9

Metric/Segmentation	Our Approach	Baseline
ROC AUC	0.763 ± 0.056	0.710 ± 0.060
Accuracy	0.563 ± 0.059	0.512 ± 0.036
Sensitivity	0.901 ± 0.076	0.901 ± 0.052
Specificity	0.323 ± 0.075	0.234 ± 0.023
PPV$_3$	0.490 ± 0.067	0.459 ± 0.049

## Discussion

We presented an approach that combines DoG with Hessian analysis and dense regression to achieve precise MC segmentation in 2D digital mammograms. To our knowledge, this is one of the first works applying a fully convolutional architecture for MC segmentation, which permits concurrent prediction on multiple adjacent locations. The method was trained and validated on 435 mammograms from two separate datasets. The results show that our method outperforms comparable approaches that have been recently published. In the FROC analysis using the INbreast dataset, our method achieves a TPR of 0.744 at false positives per unit area of 0.4 in comparison with a TPR of 0.618 at the same level of false positives as what is reported in [9]. On the segmentation task, our approach achieves a mean IoU per image of 0.670 and IoU per object of 0.607 compared to 0.524 mean IoU per image and 0.363 IoU per object for the morphological approach presented in [24]. The addition of local data, even when coarsely annotated by a human reader, improved the performance of our method. The ability to utilize a mixture of annotations, ranging from precise segmentations of larger calcifications to point estimates representing the centroid of smaller calcifications, is a strength of our approach. As an indirect validation of our method, we conducted segmentation-based MC malignancy classification. In this downstream task, our method outperformed the baseline segmentation method with a ROC AUC of 0.763 versus 0.710. Also, our approach demonstrated incremental performance in terms of its constituents (i.e., the Hessian DoG blob segmentation and the regression model). We also showed the ability to generalize our approach to other mammographic views (e.g., magnification views).

While we achieved a lower number of false positives than other approaches, the overall number of false positives per image is still high. Our approach would benefit from a false positive reduction step. Most false positives occur near larger calcifications and correspond to more irregular shapes than actual MCs. The irregular detection can be attributed to the regression model, designed to segment regions containing calcifications. In the case of larger calcifications, the segmented regions span larger areas, increasing the likelihood of retaining false positive objects. Additional filtering based on size and shape criteria in areas where large calcifications are identified could lead to a substantial false positive reduction. Human annotation and confirmation of every MC on an image is an impractical task, and algorithms should emphasize identifying MCs that are at the highest risk of being associated with cancerous lesions. Moreover, our algorithm likely identified MCs missed by human readers, inflating the false positive count. Our approach also under-segments or over-segments in certain scenarios. Undersegmentation occurs most often in large objects due to: (1) interior regions of objects having lower intensities that are omitted and (2) incorrect delineation of boundaries due to subtle contrast differences between the MC and surrounding tissue. Nevertheless, large calcifications are typically considered benign and not clinically significant. Their undersegmentation will not affect quantitative features that may predict invasive cancers. Oversegmentation occurs primarily when bright objects identified with Hessian DoG are close together and erroneously combined into a single object when only part corresponds to an actual MC.

Several limitations of our approach exist. Labeling all MCs in full-field mammograms is time-consuming and prone to human error and inter-annotator variability. Hence, our work is limited by the dataset size and variations in how MCs are annotated, ranging from point-like annotations to detailed contours. Using a proximity function to reflect the uncertainty associated with MC annotations makes our approach robust to training data variations. Moreover, the inherent differences in mammograms acquired with equipment manufactured by different vendors present another challenge. The local dataset was obtained using equipment manufactured by Hologic, whereas the public dataset INbreast was obtained using Siemens equipment. The brightness and contrast levels of the images varied substantially between manufacturers. Given that the INbreast dataset had four times as many cases as the local dataset, our model was fine-tuned with a limited number of training patches. The method was trained and evaluated using existing data prone to selection bias, making the model susceptible to underspecification. Moreover, with the increased adoption of digital breast tomosynthesis, our method has not yet been evaluated on these scans. Ongoing work includes annotating additional cases from our institution that would allow us to fine-tune the model further and experiment with different training strategies to improve the generalizability of our approach.

## Conclusions

We described a new quantitative approach for MC segmentation based on blob segmentation and dense regression. We showed that our method performs better than state-of-the-art MC segmentation and detection methods. In our case study, we evaluated the effect of the segmentation method on computed quantitative image features and classification performance. Our results suggested that our method has the potential to segment calcifications on a variety of images (FFDMs, magnification views) with minimal fine-tuning. Moreover, our method exhibited better performance than features generated using a comparison segmentation method. The case study also demonstrated the potential of quantitative characterization of MCs in improving the management of women with amorphous calcifications. Shape, intensity, and texture features can be extracted from individually segmented MCs to yield quantitative descriptors of MC morphology and distribution. While further studies are needed to evaluate the PPV_3_ and reproducibility of our quantitative features, these features, enabled by accurate segmentation of MCs, may provide a basis for reducing false positives and unnecessary biopsies.

## Supplementary Information

Below is the link to the electronic supplementary material.

Supplementary file1 (DOCX 518 KB)

## Data Availability

Our code (including trained models) is publicly available here: https://github.com/cmarasinou/HDoGReg.

## Footnotes

1 PPV_3_ is the proportion of cases that underwent biopsy due to abnormal breast imaging findings which resulted in a breast cancer diagnosis.

2 MC objects annotated by a single pixel were disregarded in computing IoU per MC object. IoU is not well defined in such cases.

3 Centroids of individual objects were used in computing their distance.
