# Supplementary material for: Improving the Quantitative Analysis of Breast Microcalcifications: A Multiscale Approach
Source: J Digit Imaging. 2023 Feb 23;36(3):1016–28. doi: 10.1007/s10278-022-00751-3 (PMC10287598; doi:10.1007/s10278-022-00751-3)
Supplement: Supplementary file 1 — Supplementary file1 (DOCX 518 KB) [file 10278_2022_751_MOESM1_ESM.pdf]

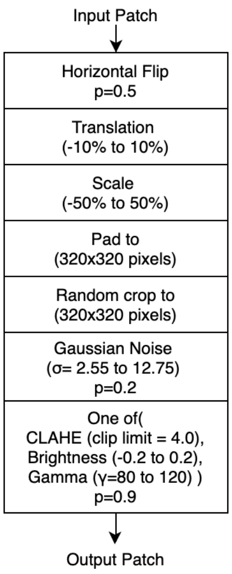

Fig. S.1 The order of augmentations applied during training.

Supplementary Information

S.1 Inter-reader reliability

MC locations were annotated in the local dataset. As described in section II, a two-reader study was conducted using a random sample of five cases (seven full-field digital mammography images) annotated by two board-certified breast radiologists. Objects between the two annotations were defined as a match if their distance was at most 5 pixels (0.35 mm). We denote the number of matched objects as a, the number of objects detected by only the first reader as b and by only the second reader as c. An appropriate reader agreement measure is the index of specific agreement (ISA) [1],

$$\mathrm{ISA} = \frac{2a}{2a+b+c} \tag{1}$$

ISA does not assume the existence of ground truth locations for the study objects. On the other hand, the kappa statistic assumes that such a ground truth exists, and therefore objects undetected by all readers are treated as objects scored negative. For completeness, we report both ISA and kappa statistics. For the latter, the number of objects undetected by both readers was set to zero. To compute the confidence intervals for both scores, bootstrap resampling was used. We generated 1000 bootstrap samples, each consisting of 7 images drawn from the original sample with replacement. For each statistic, the median value is reported along with the 95% confidence intervals. The results were:

$$\mathrm{ISA} = 0.664\ (0.606, 0.729) \tag{2}$$

$$\kappa = -0.188\ (-0.281, -0.064) \tag{3}$$

S.2 Training Augmentations

Fig. S.1 illustrates various augmentations performed on the image patches during training. All augmentations up to the random cropping step were applied concurrently to the image and the mask. The remaining steps were applied only on the image patch since they altered the greyscale levels. The probability of performing the transformation is denoted by p. The operating parameters of a given transformation are shown in parentheses. A parameter value was selected based on the uniform distribution when a range was considered.

S.3 Context-Sensitive Deep Learning Detection Implementation

This is a detailed description of the implementation of the approach presented in [2]. The method consists of two stages. First, DoG blob detection is applied to find candidate locations, and then a convolutional neural network is used to classify them into MC and non-MC locations.

We implement DoG based on the reported parameters adjusting the scales to the resolution of our dataset. Specifically, we set $\sigma_{\text{min}} = 1.6$ and $\sigma_{\text{max}} = 2.0$ at resolution 0.07 mm/pixel (in [2] $\sigma_{\text{min}} = 1.1$ and $\sigma_{\text{max}} = 1.4$ at resolution 0.10 mm/pixel). The DoG decision threshold was set to 0.01, which achieves a TPR of 0.85 at 51.5 false positives per unit area (cm^2) on the validation set.

In the second stage, we implemented the finalized convolutional network in [2], where we assume that the dropout technique is applied only before the final fully-connected layer during training. Then, we pre-processed the mammograms with a background subtraction step (implemented by subtracting the average intensity of a circular area with a diameter of 7 pixels from each location's intensity), followed by normalizing the image to zero mean and unit standard deviation. For model training, we extracted 95x95 patches centered on each annotated MC on the INbreast training set. We extracted 3481 MC samples in total. Similarly, we extracted 69620 non-MC samples from random background tissue locations (20 times as many non-MC samples as MC samples from each mammogram). Applying the augmentations in Wang et al. [2], we increased our training samples by a factor of 6. To train the model, we used the Adam optimization method with mini-batch size 256, $\beta_1 = 0.9$, $\beta_2 = 0.999$, $\epsilon = 10^{-8}$. Although the learning rate and weight decay values are not mentioned in [2], we set the learning rate to 0.001 and weight decay to 0. We follow the two-stage training strategy with 10,000 iterations of balanced mini-batches and an additional 10,000 iterations of randomly selected mini-batches.

To predict the MC locations on whole images, candidates were detected using the DoG blob detection. Then, 95x95 patches are extracted centered on each candidate location. For each patch, the inference is performed using the trained network, which outputs a probability that the location is an MC. An operating threshold is applied on all samples to retain the MC samples.

S.4 MC Malignancy Classification Tuning

Downstream malignancy classification based on MC segmentation was performed. Our method's parameters were selected based on the combination that achieved the highest ROC AUC. The best configuration was found using a grid search approach with the following values: $\sigma_{\text{min}} = \{1, 2, 3\}$, $\sigma_{\text{max}} = \{4, 5\}$, overlapping fraction $O_{\text{DoG}} = 1$, DoG threshold $T_{\text{DoG}} = \{0.05, 0.01, 0.002\}$ and Hessian threshold $h_{\text{thr}} = \{0, 0.5, 1, 1.5\}$. The grid range was decided based on qualitative experiments. As presented in Fig. S.2, the highest performance was achieved for the HDoG parameters: $\sigma_{\text{min}} = 2$, $\sigma_{\text{max}} = 4$ or 5, $T_{\text{DoG}} = 0.002$ and Hessian threshold $h_{\text{thr}} = 0.5$.

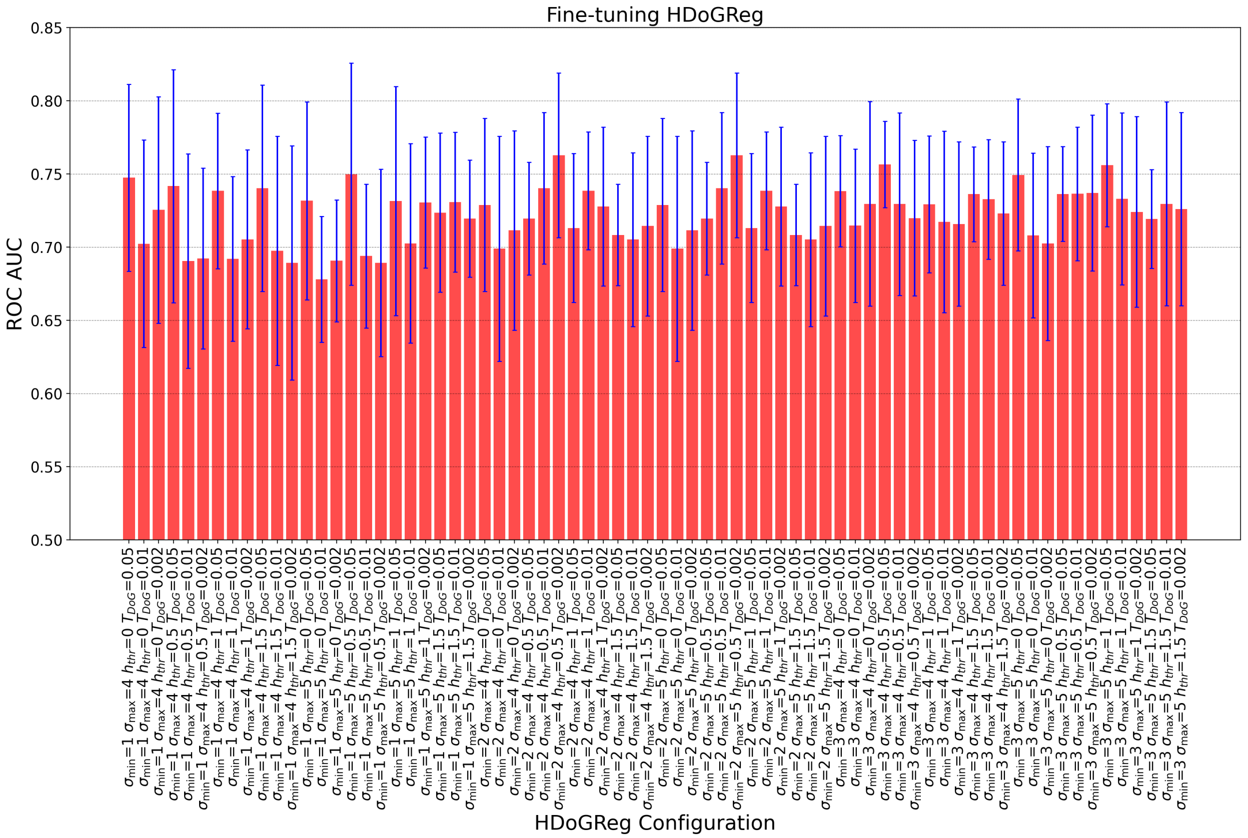

Fig. S.2 Fine-tuning our approach for the classification task. A parameter grid search is applied with the configurations shown on the x-axis. For each configuration, the mean ROC AUC (red bar) and corresponding standard deviation (error bar) are presented.

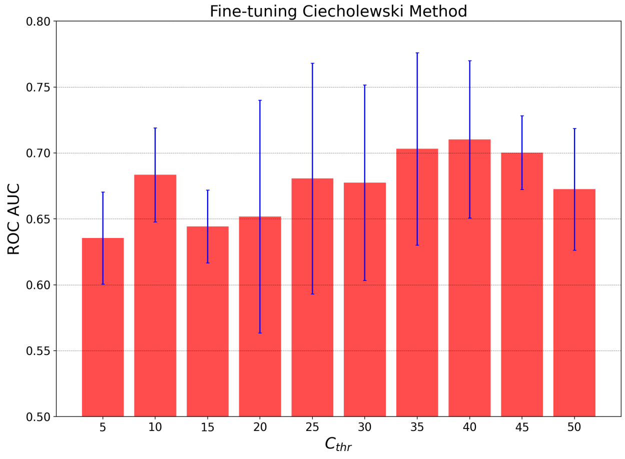

Fig. S.3 Fine-tuning the baseline segmentation method for the classification task. A single parameter, C_{thr} controlling the number of segmented MCs is tuned. For each C_{thr} considered, the mean ROC AUC (red bar) and corresponding standard deviation (error bar) are presented.

Similarly, for the baseline method, a parameter controlling the strength of false positive reduction is fine-tuned, i.e., the parameter is referred to as "Th" in the original paper (see Table 1 therein). We denote the parameter as C_{thr}. More segmented objects are removed at higher values of $C_{\text{thr}} \in [0, 255]$. The highest ROC AUC is achieved at $C_{\text{thr}} = 40$ (see Fig. S.3).

References

[1] Shih JH, Greer MD, Turkbey B. The Problems with the Kappa Statistic as a Metric of Interobserver Agreement on Lesion Detection Using a Third-reader Approach When Locations Are Not Prespecified. Acad Radiol. 2018;25(10):1325-32.

[2] Wang J, Yang Y. A context-sensitive deep learning approach for microcalcification detection in mammograms. Pattern Recognition. 2018;78:12 22.
